# Relationships between postpartum depression, sleep, and infant feeding in the early postpartum: An exploratory analysis

**DOI:** 10.3389/fpsyt.2023.1133386

**Published:** 2023-03-24

**Authors:** Alanna E. F. Rudzik, Lyn Robinson-Smith, Francesca Tugwell, Helen L. Ball

**Affiliations:** ^1^Durham Infancy and Sleep Centre, Durham University, Durham, United Kingdom; ^2^Department of Anthropology, Durham University, Durham, United Kingdom

**Keywords:** postpartum depression, maternal sleep, infant sleep, infant feeding, postpartum period, women’s mental health

## Abstract

**Introduction:**

The study objectives were to determine the relationships between postpartum depression and maternal and infant sleep parameters and to examine the impact of infant feeding method on infant and maternal sleep and postpartum depression symptomatology.

**Methods:**

Participants were 61 new mothers aged 18 to 45 years old, and their full-term, normal birth-weight, singleton infants. Participants were recruited from a large teaching hospital in northeast England. Data collection took place in participants’ homes. The study used a prospective longitudinal design, with data collected at six, 12 and 18 weeks postpartum. We collected data on total sleep time, longest sleep period, wake after sleep onset, and night waking for mothers and infants objectively from actigraphic records and subjectively from maternal sleep logs. Participants reported on sleep disturbances using the General Sleep Disturbances Scale, on maternal sleepiness, and on depression symptomatology using the Edinburgh Postnatal Depression Scale.

**Results:**

Scores on the Edinburgh Postnatal Depression Scale and General Sleep Disturbances Scale were consistently correlated with each other (6 weeks r = 0.452, *p* < 0.01; 12 weeks *r* = 0.317, *p* < 0.05; 18 weeks *r* = 0.493, *p* < 0.01), and did not correlate with objective measures or subjective reports of maternal or infant sleep. Edinburgh Postnatal Depression Scale scores at six, 12 and 18 weeks were predicted by General Sleep Disturbances Scale, prior Edinburgh Postnatal Depression Scale score, or both, but not by sleep parameters. With regard to infant feeding method, EPDS score was not higher among exclusively breastfeeding than among exclusively formula-feeding participants at any time point (6 weeks  *t =* 0.306, *p* = 0.762; 12 weeks  *t =* 0.343, *p* = 0.733; 18 weeks  *t =* 0.426; *p* = 0.673). Different pathways emerged to predict Edinburgh Postnatal Depression Scale score for exclusively breastfeeding and exclusively formula-feeding women.

**Discussion:**

Postpartum depression may be associated with disturbed sleep due to negative perception of sleep among depressed women, rather than disrupted sleep causing postpartum depression. With regard to infant feeding method, exclusively breastfeeding women are not more likely to suffer from postpartum depression, and different pathways may predict development of postpartum depression symptoms in exclusively breastfeeding and exclusively formula feeding women.

## 1. Introduction

The early postpartum is a time of major transition for new parents. When an infant is brought into the home, parents must adjust to new roles and changes to their day to day lives, including—and perhaps most notably—with regard to sleep. Mood disorders are prevalent in the postpartum period with 30–75% of new mothers experiencing at least mild and transient mood disturbances in the first few days postpartum (1). Ten to 20 % of United Kingdom parents are diagnosed with postpartum depression clinically (2, 3), while up to a further 50% of parents experience mild to moderate postpartum depression symptoms (4). Postpartum depression can occur any time within the first year postpartum and can last anywhere from 2 weeks to 2 years (5). Postpartum depression is associated with poverty, stress, inadequate social support and interpersonal conflict (6).

Postpartum depression is detrimental to parents and infants (7), resulting in lower personal, household, and social functioning (5); increased social withdrawal and family dysfunction (8); reduced enjoyment of the new baby, increased family distress (9); and increased risk of future depressive episodes for both parents (10). Parents with severe postpartum depression are at increased risk for suboptimal caregiving, potentially affecting infant cognitive and developmental outcomes (7, 9). Women with depression are less likely to breastfeed (11), reducing access to the short-and long-term benefits breastfeeding confers.

Poor maternal sleep has been widely linked to the development of postpartum depression and depression symptom severity (12–14). Infant-related sleep disruption has shown a strong and enduring association with maternal depression (12, 15) even when accounting for recognized depression risk factors (16). However, recent findings have cast doubt on the assumed direction of causality (disrupted sleep leads to increased depression) (17–20). Additionally, subjective parental assessments of infant sleep, which in many studies are used alone to assess infant sleep, show variable accuracy when compared with data collected using objective sleep measures (21–23). Depression is one of the factors that affects the accuracy of parental reports of infant sleep (20). Women with worse mental health report increased night waking and bedtime distress on the part of their infants, are more bothered by these sleep issues, and more often seek treatment for their infants’ perceived sleep problems (24, 25). Yet many researchers have suggested that if sleep in the postpartum period contributes to the development and extent of postpartum depression symptoms, modifying infant sleep may reduce maternal risk of postpartum depression (26, 27). Multiple family-based sleep interventions are rationalized based on this assumption (16, 28, 29). Assumptions that breastfeeding mothers are particularly prone to poor sleep, as the result of night-time feeding, have been incorporated into this discourse (30). A switch to formula, in order to protect maternal sleep has been proposed as a type of maternal “self-care” that may help prevent the development of postpartum depression (31).

In this paper, we examine the associations between (1) maternal and infant sleep as recorded objectively using actigraphy; (2) maternal and infant sleep as reported by participants; (3) sleep disturbance reported retrospectively; (4) experience of daytime sleepiness; and (5) depression symptomatology, in order to elucidate the relationship between postpartum depression and maternal and infant sleep. In addition, we investigate the impact of infant feeding method on sleep measures and reports, sleep disturbance, sleepiness and depression symptomatology, to determine whether differences exist with regard to sleep parameters, sleep disturbance, sleepiness, and predictors of postpartum depression between women who exclusively breastfeed and those who exclusively formula-feed.

## 2. Materials and methods

### 2.1. Participant inclusion and exclusion criteria

Primiparous and multiparous mothers of healthy singleton newborns, full-term and of normal birthweight, were contacted on the postnatal ward of a large teaching hospital in the northeast of England between September 2012 and December 2013. Women between 18 and 45 years of age who intended to breastfeed or formula-feed exclusively for the first 18 weeks postpartum were invited to participate, along with their infants. Women who gave birth to preterm, low birth-weight, or ill infants were excluded from participation, as were women who had multiple births or were under 18 years of age or over 45 years of age. Because infant feeding method was a variable of particular interest, women who did not express a clear intention to either breastfeed exclusively or formula feed exclusively through the period of study (4–18 weeks postpartum) were also excluded from participation.

Those who expressed an initial interest in participating in the study were provided with an informational leaflet and gave permission for a telephone follow-up at two weeks postpartum, at which time they were asked whether they wished to participate in the study. Of 283 women who agreed to be contacted, 61 ultimately agreed to participate along with their infants (response rate = 22%) and scheduled a first data collection visit. Participants provided written informed consent at the first visit, prior to any data collection occurring. Ethics approval for the study was granted by Durham University and the UK National Health Service Research Ethics Committee.

### 2.2. Data collection

Participants completed a demographic questionnaire, including data about ethnicity, participant’s location of birth, marital status, and household income during a home visit at 4 weeks postpartum. All data were collected in participants’ homes, to minimize the disruption to sleep and other behaviors that may arise from involvement in a study as such an early period postpartum. The design of the larger study involved data collection every two weeks between four and 18 weeks postpartum (eight time points in total) of a wide range of objective, subjective and biological data. This paper reports on data collected at the six, 12 and 18 week visits (three time points), as described below.

#### 2.2.1. Self-report measures of sleep disturbance, sleepiness, and depression

Participants completed measures that assessed sleep disturbance, sleepiness, and depression symptomatology. The General Sleep Disturbances Scale (GSDS), developed by KA Lee (32), is a retrospective self-report scale used to assess sleep disturbance. Respondents are asked to respond to each question thinking about the last 7 days. The GSDS shows internal consistency ranging from 0.77 to 0.85 (33) and has been validated for use with new parents. The measure was self-administered in hardcopy, taking 5–10 min to complete.

A pictorial scale based on cartoon faces, as developed by Maldonado et al. (34), was used to evaluate participants’ sleepiness in a variety of different situations. Participants were asked to think of their experience in recent days when responding. The initial validation of the sleepiness measure showed high correlation with other sleepiness scales (Stanford, Karolinska) and virtually all participants (99%) were able to correctly rank the sleepiness of the cartoon faces (34). The measure was self-administered in hardcopy, taking less than 3 min to complete.

The Edinburgh Postnatal Depression Scale (EPDS) was used to assess postpartum depression risk. The scale was developed by Cox and colleagues and shows good validity (sensitivity 86%; specificity 78%) (35). The scale was self-administered in hardcopy, taking about 5 min to complete. A score of 12 or higher indicates risk of a serious depressive illness (35), thus we categorized those who scored 12 or higher as being at risk of postpartum depression. As part of our ethical obligation to safeguard participants, we notified participants in advance that we would contact the GP (primary care physician) of any participant who, at any point during the study, scored at or above the cut-off of 12 and/or who validated the question related to suicidal ideation. We followed this protocol throughout the study as the need arose.

#### 2.2.2. Objective measures of maternal and infant sleep

Objective measures of maternal and infant sleep were obtained through the use of Micro Motionlogger actigraphic watches (Ambulatory Monitoring Inc., Ardsley, NY). While sleep polysomnography is considered the “gold standard” for objective sleep measurement there are both practical and ethical considerations that make actigraphy preferable for data collection at multiple time points and in naturalistic (i.e., home-based) settings (36). Participants were shown how to position the watches correctly during the first home visit; infants’ watches were worn on the left thigh and mothers’ watches were worn on the non-dominant wrist. For subsequent data collection, a researcher dropped off the watches with the participants during the day prior to the overnight data collection. Participants (mothers and infants) then wore the watches overnight for one night at each time point (six, 12 and 18 weeks). The overnight period of interest was specified as 6 pm to 8 am. The watches were pre-programmed to begin recording at 6 pm and to continue recording, using a one-minute epoch interval for sleep–wake scoring, until the watches were collected by the research team and the data uploaded.

Data records for each recording were cleaned by the researchers to ensure correct scoring of sleep/wake, making reference to temperature and light channels, as well as movement, for accuracy. For example, periods between 6 pm and 8 am when watches were removed (e.g., for bathing) were identified using data from the watches’ temperature channel, combined with identification of clearly anomalous activity periods (i.e., abrupt zero-movement periods followed by resumption of usual movement).

Infant and maternal actigraphic sleep data were processed using Sadeh’s scoring algorithms for infant and adult sleep, respectively (37, 38). The software automatically calculated maternal total sleep time (TST), and maternal and infant overnight longest sleep period (LSP), number of night wake episodes (NW), and wake after sleep onset (WASO), and these parameters were exported. Objective infant overnight TST was calculated by adding the total number of epochs scored as sleep or light sleep, as exported from the software.

#### 2.2.3. Subjective reports of maternal and infant sleep

Subjective reports of maternal and infant sleep/wake were collected by having participants complete a hardcopy sleep log on the same night as actigraphic data were collected and covering the same overnight period (6 p.m.–8 a.m.). The sleep logs divided the overnight period into 15-min blocks, with separate columns for infant and maternal sleep. Participants were instructed to indicate within each 15-min block when sleep occurred using a check mark, shading or other indication. Participants were asked to complete the sleep log each time that they awakened (including overnight wakings), to minimize recall error.

Subjective maternal and infant sleep parameters were obtained from the hardcopy sleep logs. Subjective overnight TST was calculated by adding all 15-min periods between 6 pm and 8 am during which the participant indicated that they/the infant were sleeping. Subjective night-time LSP was calculated by adding the longest continuous set of 15-min blocks during which the participant indicated that they/the infant were sleeping. Subjective number of night wakings was calculated by counting the periods of wakefulness occurring between the initial onset of sleep and the final 15-min period of sleep. Subjective WASO was calculated by adding all 15-min blocks of wake time that occurred after initial onset of night-time sleep and before the final 15-min period of sleep.

#### 2.2.4. Infant feeding categories

Infant feeding method was obtained from participants for each infant at each data collection visit *via* a hardcopy survey instrument. In separate questions, participants were asked to indicate which type of food their infant *predominantly* consumed and to indicate *all* the foods their infant consumed. Only infants whose mothers indicated sole consumption of human milk or sole consumption of formula milk were classified as exclusively breastfeeding or exclusively formula-feeding, respectively. Infants who consumed a mix of human milk and formula milk, formula and solids, or human milk and solids were all categorized as mixed feeding. Data from infants who were reported to be mixed feeding were excluded from the feeding-related analyses below (*n* = 36 observations).

### 2.3. Data analysis

All statistical analyses were conducted using SPSS version 22.0 (IBM Corp., Armonk, NY, United States). Descriptive statistics were computed for sociodemographic characteristics. Chi-square and *t*-test analyses were used to determine whether there were statistical differences in the underlying sociodemographic characteristics of exclusively breastfeeding and exclusively formula-feeding participants.

Correlation analyses were run for the sleep parameters recorded objectively using actigraphy, those collected subjectively from sleep diaries, sleep disturbance as rated on the GSDS, daytime sleepiness scale score, and postpartum depression symptomatology using the EPDS. Linear regression models predicting EPDS score at six, 12 and 18 weeks were constructed on the basis of the bivariate correlation analyses. Variables were entered into the regression model in blocks, using a stepwise method. The first block consisted of variables that were significantly correlated with EPDS score for the relevant time point. The second block consisted of variables that approached significance (0.05 < *p* < 0.10). The third block included maternal age and household income (above/below UK median). Cases with missing data were excluded listwise.

The bi-variate relationships between infant feeding method, objective and subjective sleep parameters, maternal sleep disruption and sleepiness, and risk of postpartum depression, were analyzed using *t*-tests. Subsequently, linear regression models were constructed to determine the predictors of EPDS scores for exclusively breastfeeding and exclusively formula-feeding women separately. Variables were entered by block as above and a stepwise method was again used.

## 3. Results

### 3.1. Participant sociodemographic characteristics

Our sample of participants from the northeast of England was predominantly White (92%), UK-born (88%), and married (87%). Maternal age ranged from 18 to 45 years (mean = 30.5y ±6.8y). The sample was relatively affluent, with 71% reporting household income above the UK median at the time (39). Complete sociodemographic data are given in Table 1.

**Table 1:** Participant demographic data.

Participant characteristic	Mean (S.D.)/Percentage of sample
Participant age	30.5y (18–45; *S.D.* 6.8y)
*Ethnicity*	
White	55/60 (92%)
Asian-British, Black/African/Caribbean/Black British, Mixed/Multiple Ethnic Groups	5/60 (8%)
*UK-born*	
Yes	53/60 (88%)
No	7/60 (12%)
*Marital status*	
Married/Living with partner	52/60 (87%)
Single, No partner/With partner, living apart*	8/60 (13%)
*Household Income (dichotomized)*	
Below UK 2013 median household income	17/58 (29%)
Above UK 2013 median household income	41/58 (71%)

*For Marital Status the categories “Single, No partner” and “With partner, living apart” were merged due to low case count, which might make participants identifiable.

Individuals’ EPDS scores were consistent through time. Roughly 10% of participants who completed the EPDS measure scored at or above the cut-off of 12 at each time point (6 weeks = 10.9%, 12 weeks = 9.6%, 18 weeks = 10.2%). Only one participant scored 12 or above at all three time points.

Roughly one-third of women were exclusively breastfeeding at each data-collection point, while half or slightly more were exclusively formula-feeding. The proportion of women who were using mixed feeding increased through time from 1 in 13 at 6 weeks to 1 in 5 at 18 weeks. Details of feeding method by time are shown in Table 2.

**Table 2:** Infant feeding method by time.

	6 weeks	12 weeks	18 weeks
Exclusively breastfeeding	20/53 (37.7%)	17/51 (33.3%)	16/51 (31.4%)
Exclusively formula feeding	29/53 (54.7%)	29/51 (56.9%)	25/51 (49.0%)
Mixed feeding	4/53 (7.5%)	5/51 (9.8%)	10/51 (19.6%)

There was no association between household income and feeding method at any time point (6 weeks: *χ* = 0.739, *p* = 0.390; 12 weeks: *χ* = 0.024, *p* = 0.874; 18 weeks: *χ* = 0.005, *p* = 0.942) and there was no significant difference in maternal age between exclusively breastfeeding and exclusively formula-feeding participants (*t* = 0.741, *p* = 0.463).

### 3.2. Analysis of relationships between sleep and postpartum depression

The key variables in our analysis were (a) subjective reports and objective measures of maternal and infant sleep parameters (TST, LSP, WASO, night waking); (b) self-report measures of sleep disturbance (GSDS) and maternal sleepiness and (c) self-report of postpartum depression symptomatology (EPDS).

#### 3.2.1. Bivariate analyses

GSDS score did not correlate with subjective or objective TST, WASO, or NW measures or reports of infant or maternal sleep; it correlated with subjective report of maternal and infant LSP at 18 weeks only (maternal: *r* = −0.334, *p* < 0.05; infant: *r* = −0.322, *p* < 0.05). GSDS score correlated strongly with same-week sleepiness scores at all time points (6 weeks r = 0.618, *p* < 0.01; 12 weeks *r* = 0.665, *p* < 0.01; 18 weeks *r* = 0.549, *p* < 0.01). At six, 12 and 18 weeks EPDS scores correlated with different variables. At 6 weeks, none of the objective or subjective maternal or infant sleep measures were significantly correlated with 6-week EPDS score, whereas 6-week GSDS (*r* = 0.452, *p* < 0.01) and 6-week sleepiness scores (*r* = 0.445, *p* < 0.01) were both correlated with 6-week EPDS score. At 12 weeks, subjectively reported infant night waking frequency (*r* = 0.340, *p* < 0.05) was significantly correlated with 12-week EPDS, as were 6-week EPDS (*r* = 0.605, *p* < 0.01) and 12-week GSDS (*r* = 0.317, *p* < 0.05). At 18 weeks, none of the subjective or objective maternal or infant sleep measures were significantly correlated with 18-week EPDS; only 12-week EPDS (*r* = 0.565, *p* < 0.01) and 18-week GSDS scores (*r* = 0.493, *p* < 0.01) were significantly correlated with 18-week EPDS. The full correlations are shown in Tables 3–5.

**Table 3:** Bivariate correlations between sleep measures, self-report measures, and EPDS at 6 weeks.

Correlations																		
		1	2	3	4	5	6	7	8	9	10	11	12	13	14	15	16	17
1	EPDS6	1																
2	GSDS6	0.452**	1															
3	Sleepiness6	0.445**	0.618**	1														
4	Mat Obj TST 6wk	−0.261	−0.089	−0.075	1													
5	Mat Obj LSP 6wk	−0.037	0.086	0.075	0.083	1												
6	Mat Obj WASO 6wk	−0.025	0.137	0.076	0.091	−0.419**	1											
7	Mat Obj NW 6wk	−0.129	−0.176	−0.112	0.351**	−0.685**	0.396**	1										
8	Inf Obj TST 6wk	−0.126	0.004	−0.087	0.439**	−0.027	0.280*	0.337*	1									
9	Inf Obj LSP 6wk	0.219	0.103	0.045	0.204	0.160	−0.013	−0.038	0.276	1								
10	Inf Obj NW 6wk	−0.076	−0.058	0.106	0.158	0.078	0.089	−0.012	0.280*	−0.339*	1							
11	Inf Obj WASO 6wk	−0.028	0.211	0.248	−0.270	0.234	0.023	−0.236	−0.065	−0.242	0.010	1						
12	Mat Subj TST 6wk	0.072	0.099	0.056	0.467**	0.137	−0.078	0.207	0.206	0.298*	0.095	−0.220	1					
13	Mat Subj LSP 6wk	0.275	0.231	0.182	0.253	0.279	−0.340*	−0.071	0.129	0.274	−0.010	0.109	0.587**	1				
14	Inf Subj TST 6wk	0.075	−0.245	−0.137	0.259	−0.132	−0.057	0.292*	0.153	0.038	0.104	−0.203	0.439**	0.212	1			
15	Inf Subj LSP 6wk	0.253	−0.247	0.039	0.155	0.130	−0.299*	0.060	0.161	0.305*	0.015	−0.143	0.347*	0.496**	0.587**	1		
16	Inf Subj NW 6wk	−0.130	−0.020	−0.148	−0.050	−0.142	0.083	0.103	−0.073	−0.380**	−0.024	0.169	−0.148	−0.211	0.093	−0.320*	1	
17	Inf Subj WASO 6wk	−0.251	0.129	−0.060	−0.269	0.077	−0.064	−0.274	−0.221	−0.170	−0.164	0.308*	−0.514**	−0.276	−0.586**	−0.458**	0.277*	1

**Correlation is significant at the 0.01 level (2-tailed).

*Correlation is significant at the 0.05 level (2-tailed).

**Table 4:** Bivariate correlations between sleep measures, self-report measures, and EPDS at 12 weeks.

Correlations																		
		1	2	3	4	5	6	7	8	9	10	11	12	13	14	15	16	17
1	EPDS12	1																
2	GSDS12	0.317*	1															
3	Sleepiness12	0.068	0.665**	1														
4	Mat Obj TST 12wk	0.015	−0.065	0.003	1													
5	Mat Obj LSP 12wk	−0.064	0.032	0.036	0.546**	1												
6	Mat Obj WASO 12wk	0.024	−0.197	−0.059	0.031	−0.370*	1											
7	Mat Obj NW 12wk	0.026	−0.030	0.164	0.099	−0.596**	0.650**	1										
8	Inf Obj TST 12wk	0.178	0.111	0.153	0.282	0.277	−0.286	−0.131	1									
9	Inf Obj LSP 12wk	0.056	0.277	0.240	0.169	0.265	−0.210	−0.206	0.522**	1								
10	Inf Obj NW 12wk	−0.165	−0.113	−0.059	0.166	−0.044	0.088	0.221	−0.247	−0.526**	1							
11	Inf Obj WASO 12wk	0.020	0.073	0.096	0.294	0.063	0.029	0.123	−0.276	−0.142	0.378*	1						
12	Mat Subj TST 12wk	−0.168	−0.059	−0.007	0.841**	0.521**	−0.140	−0.054	0.252	0.194	0.074	0.060	1					
13	Mat Subj LSP 12wk	−0.184	−0.050	0.017	0.513**	0.647**	−0.309*	−0.291	0.097	0.162	0.103	0.133	0.671**	1				
14	Inf Subj TST 12wk	−0.169	−0.035	0.073	0.433**	0.394**	−0.225	−0.041	0.392*	0.109	0.265	0.055	0.295*	0.416**	1			
15	Inf Subj LSP 12wk	−0.188	−0.112	−0.045	0.229	0.348*	−0.201	−0.157	−0.142	−0.133	0.371*	0.117	0.235	0.625**	0.572**	1		
16	Inf Subj NW 12wk	0.340*	0.082	0.072	−0.174	−0.134	0.183	0.091	0.095	0.245	−0.465**	−0.034	−0.241	−0.446**	−0.343*	−0.752**	1	
17	Inf Subj WASO 12wk	0.108	−0.042	−0.085	−0.195	−0.005	0.144	−0.175	−0.306	0.133	−0.310*	0.138	−0.195	−0.159	−0.453**	−0.423**	0.668**	1

*Correlation is significant at the 0.05 level (2-tailed).

**Correlation is significant at the 0.01 level (2-tailed).

**Table 5:** Bivariate correlations between sleep measures, self-report measures, and EPDS at 18 weeks.

Correlations																		
		1	2	3	4	5	6	7	8	9	10	11	12	13	14	15	16	17
1	EPDS18	1																
2	GSDS18	0.493**	1															
3	Sleepiness18	−0.014	0.549**	1														
4	Mat Obj TST 18wk	−0.149	−0.187	−0.138	1													
5	Mat Obj LSP 18wk	−0.017	−0.051	0.053	0.210	1												
6	Mat Obj WASO 18wk	−0.145	−0.233	−0.138	0.027	−0.492**	1											
7	Mat Obj NW 18wk	−0.127	−0.173	−0.072	0.124	−0.740**	0.637**	1										
8	Inf Obj TST 18wk	−0.107	−0.047	0.094	0.131	−0.119	−0.259	0.119	1									
9	Inf Obj LSP 18wk	−0.185	−0.172	−0.104	−0.110	0.047	−0.128	0.010	0.426**	1								
10	Inf Obj NW 18wk	0.013	0.044	0.033	0.211	0.053	0.005	−0.023	0.178	−0.497**	1							
11	Inf Obj WASO 18wk	0.093	−0.050	−0.033	−0.336*	−0.101	0.238	−0.018	−0.025	−0.166	0.259	1						
12	Mat Subj TST 18wk	−0.028	−0.093	−0.043	0.568**	0.048	−0.352*	0.059	0.285	0.101	−0.002	−0.326*	1					
13	Mat Subj LSP 18wk	−0.246	−0.334*	−0.114	0.155	0.408**	−0.418**	−0.290	−0.012	0.097	−0.085	−0.097	0.510**	1				
14	Inf Subj TST 18wk	−0.249	−0.060	−0.066	0.367*	−0.006	−0.146	0.128	0.525**	0.170	0.082	−0.334*	0.332*	0.151	1			
15	Inf Subj LSP 18wk	−0.273	−0.322*	−0.133	0.205	0.255	−0.157	−0.018	0.119	0.019	0.032	−0.278	0.200	0.576**	0.617**	1		
16	Inf Subj NW 18wk	0.036	0.057	0.068	−0.013	−0.352*	0.202	0.182	0.123	0.232	−0.233	0.192	−0.005	−0.414**	−0.085	−0.579**	1	
17	Inf Subj WASO 18wk	−0.073	−0.125	0.056	−0.030	−0.302*	0.302	0.269	−0.055	0.126	−0.236	0.233	−0.135	−0.184	−0.485**	−0.425**	0.486**	1

**Correlation is significant at the 0.01 level (2-tailed).

*Correlation is significant at the 0.05 level (2-tailed).

#### 3.2.2. Linear regression models

Predictors of EPDS score varied through time. At 6 weeks, EPDS score was predicted only by 6-week GSDS score (β = 0.084, 95%CI 0.031–0.137; *p* = 0.002). No other variables reached the significance threshold to be included in the model. The model was highly significant but explained only about 17% of the variation in 6-week EPDS score (*n* = 46; *p* = 0.002, Adj *R*^2^ = 0.174). At 12 weeks, EPDS score was predicted only by 6-week EPDS score (β = 0.652, 95%CI 0.388–0.875; *p* < 0.001), which explained more than one-third of the total variation (*n* = 43; *p* < 0.001; Adj *R*^2^ = 0.385). At 18 weeks, EPDS score was predicted by both 18-week GSDS score (β = 0.533, 95%CI 0.071–0.179; *p* < 0.001) and 12-week EPDS score (β = 0.350, 95%CI 0.150–0.724; *p* = 0.004). The model was highly significant and explained nearly half the variation in 18-week EPDS scores (*n* = 44; *p* < 0.001; Adj *R*^2^ = 0.479). None of the objective measures or subjective reports of infant or maternal sleep were significant predictors of EPDS score at any of the three time points.

### 3.3. Analysis of relationship between sleep and depression by infant feeding method

#### 3.3.1. Bivariate analyses

In *t*-tests, no significant differences were found between exclusively breastfeeding and exclusively formula-feeding women in EPDS score (6 weeks *t* = 0.306 *p* = 0.762; 12 weeks *t* = 0.343, *p* = 0.733; 18 weeks *t* = 0.426, *p* = 0.673), GSDS score (6 weeks *t* = 0.997, *p* = 0.380; 12 weeks *t* = 0.911, *p* = 0.367; 18 weeks *t* = 1.114; *p* = 0.273), or sleepiness score (6 weeks *t* = −0.409, *p* = 0.685; 12 weeks *t* = −0.400, *p* = 0.680, 18 weeks *t* = 0.555, *p* = 0.582) at any of the three time points. Full details are shown in Table 6.

**Table 6:** *T*-test comparisons of EPDS, GSDS and sleepiness score for exclusively breastfeeding and exclusively formula feeding women.

		Exclusively breastfeeding women			Exclusively formula feeding women					
		*n*	Mean	S.D.	*n*	Mean	S.D.	*t* value	d.f.	*p*
6 weeks	EPDS	19	4.84	3.934	29	4.48	4.050	0.306	46	0.762
	GSDS	19	45.32	21.226	27	40.11	18.375	0.997	44	0.380
	Sleepiness	19	13.32	4.282	27	5.413	1.042	−0.409	44	0.685
12 weeks	EPDS	17	4.00	4.198	27	3.59	3.598	0.343	42	0.733
	GSDS	17	37.06	19.763	27	31.85	17.611	0.911	42	0.367
	Sleepiness	17	10.94	4.548	29	11.55	5.234	−0.400	44	0.680
18 weeks	EPDS	16	4.75	5.791	23	4.09	3.953	0.426	37	0.673
	GSDS	16	39.69	22.434	23	32.35	18.605	1.114	37	0.273
	Sleepiness	15	10.93	5.216	25	10.04	4.756	0.555	38	0.582

Chi-square analyses showed no difference between the two feeding groups with regard to the proportion of women scoring above and below the EPDS cut-off (12) at each time point (6 weeks: *χ* < 0.001, *p* = 0.98; 12 weeks: *χ* = 0.024, *p* = 0.624; 18 weeks: *χ* = 2.126, *p* = 0.145).

#### 3.3.2. Linear regression models

When linear regression models were constructed to determine the predictors of EPDS scores for exclusively breastfeeding and exclusively formula-feeding women separately the predictors varied by feeding method and time point. At the 6 week time point, none of the variables considered in this study predicted EPDS score for exclusively breastfeeding women, whereas for exclusively formula-feeding women 6-week sleepiness score strongly predicted 6-week EPDS score (β = 0.665, 95%CI 0.201–0.705; *p* = 0.001; Adj *R*^2^ = 0.411). At 12 weeks, only 6-week EPDS score predicted 12-week EPDS score, for both exclusively breastfeeding women (β = 0.638, 95%CI 0.207–1.508; *p* = 0.014; Adj *R*^2^ = 0.358) and exclusively formula-feeding women (β = 0.667, 95% CI 0.334–0.927; *p* < 0.001; Adj *R*^2^ = 0.422). At 18 weeks, 18-week GSDS score (β = 0.896, 95%CI 0.136–0.326; *p* < 0.001) and household income (β = −0.375, 95%CI –9.624 – −0.098; *p* = 0.046) predicted 18-week EPDS for exclusively breastfeeding women (*p* = 0.001; Adj *R*^2^ = 0.631), whereas 12-week EPDS was the only predictor of 18-week EPDS for exclusively formula-feeding women (β = 0.728, 95%CI 0.419–1.075; *p* < 0.001; Adj R^2^ = 0.506).

## 4. Discussion

Our findings offer important insight into the relationship between sleep and depression in the postpartum, as well as the impact of infant feeding method on this relationship. Our results suggest that, at least for a community sample of health women, connections between sleep and postpartum depression may not reflect actual disruption to maternal sleep (based on data from objective measures or subjective reports of maternal and infant sleep collected immediately upon awakening). Rather, women experiencing postpartum depression symptoms may experience their own and their infant’s sleep negatively, and therefore rate it poorly; this may then lead to the association between higher depression symptomatology and sleep disruption, when retrospective instruments (like the GSDS) or experiential reports (like the sleepiness scale) are employed. A second important contribution relates to infant feeding method and postpartum depression. Our community-based sample of healthy exclusively breastfeeding women did not show increased depression symptomatology or an increased risk of postpartum depression. Results from regression analyses suggest that exclusively breastfeeding and exclusively formula-feeding women may experience different pathways that predict postpartum depression symptomatology, despite having similar mean scores.

### 4.1. Sample characteristics

Our sample from the northeast of England showed little diversity in terms of ethnicity, location of birth, and marital status. The average maternal age for our sample was 30.5 years, only slightly older than the 2013 UK average of 30.0 years (39). Household income was high across the sample, with 71% of the participants reporting a household income above the UK median. As a result of our purposeful recruitment of women who intended to breastfeed exclusively for at least 18 weeks or formula-feed exclusively for the same period, our sample has a higher rate of exclusive breastfeeding compared with findings from the UK 2010 Infant feeding Survey (40). Roughly one-third of participants at each of the three time points were exclusively breastfeeding, compared with rates of 24% at 6 weeks, 17% at 3 months and 12% at 4 months for England (40). Our sample did not show the usual association between household income and infant feeding method (breastfeeding being associated with higher income). A possible explanation of this is that higher-income formula-feeding women were more likely to self-select into the study than lower-income formula-feeding women.

Individual women’s scores on the EPDS remained consistent from 6 to 18 weeks postpartum, as did the percentage of women whose scores indicated a risk for postpartum depression, at approximately 10% of participants. Petersen and colleagues report a rate of depression within the first year after birth of 11% of all primary care-registered women in the UK, for women who gave birth from 2000 to 2013 (3), which suggests that a rate of 10% is plausible.

### 4.2. Sleep and postpartum depression: Bivariate analyses

Thought-provoking findings emerged from bivariate analyses. None of the objective actigraphic measures of maternal or infant sleep correlated with depression symptom score at any time point. No subjectively reported sleep parameters for the woman or her infant correlated with depression symptomatology at six or 18 weeks. Subjectively reported infant night waking was the only sleep parameter correlated with EPDS at 12 weeks. By contrast, EPDS score correlated with the GSDS at all three time points.

Although the GSDS aims to capture the experience of sleep, scores on the instrument did not correlate with any objective measure or subjective report of maternal or infant sleep at six or 12 weeks. It correlated only with subjectively reported longest sleep period for mothers and infants at 18 weeks (but not with actigraphically measured longest sleep periods). As such, it appears that GSDS is capturing participants’ perceptions of their sleep rather than their actual sleep experience. As a retrospective measure, there is greater potential for recall bias with the GSDS than with maternal subjective assessments of sleep collected immediately upon awakening. Participants who were experiencing higher levels of postpartum depression symptoms may have been prone to assess their sleep experience of the past week more negatively than those with lower levels of symptoms. This could explain why participants’ scores on the GSDS were not correlated with their subjectively reported sleep parameters.

The consistent correlation between EPDS and GSDS scores, in the absence of a correlation with the objective measures of sleep and the subjective reports of sleep, suggests to us that.

women with higher levels of depression symptomatology may be more likely in retrospective reports to assess and report their sleep negatively in ways that do not align with their day to day sleep experience.

### 4.3. Sleep and postpartum depression: Regression analyses

Our findings provide evidence that contradicts the argument that postpartum sleep disruption has a causal relationship with postpartum depression and lend support to the idea that women who are experiencing postpartum depression may have distorted, negatively-skewed perceptions of their own and their infants’ sleep. In regression analyses, none of the objective measures or subjective reports of maternal or infant sleep predicted depression symptomatology. Rather, GSDS score was significantly predictive of depression symptomatology at six and 18 weeks. At 12 and 18 weeks the previous postpartum depression score (6-week EPDS and 12-week EPDS, respectively) was strongly predictive of EPDS score. Other researchers have reported relationships between depression and subjective perceptions of sleep deprivation and sleep quality, regardless of objective sleep measures (16). These findings have been taken to suggest that perceptions of poor sleep and an awareness of its negative effects on daytime functioning are what lead to depression symptoms, rather than the actual quality and quantity of sleep (1). We would argue that the relationship may be the reverse and that increased depression symptomatology skews women’s perceptions of sleep and influences retrospective assessment of sleep. Previous studies have reported that women with depression were more likely to report a mismatch between maternal and infant sleep (15) and that depressed mothers were more likely to experience postpartum fatigue and to perceive their infants more negatively (41). These findings suggest that maternal depression may have an important impact on the general assessment of sleep and well-being in the postpartum.

Further research on this topic is essential. Infant behavior modification courses, products and individual consultants abound, based on the assumption that infant sleep determines maternal sleep and that maternal sleep strongly predicts risk of postpartum depression. Some researchers have argued that if sleep influences the development and extent of postpartum depression symptoms, sleep in the postpartum period may be a modifiable risk factor for the development of postpartum depression (16, 28–30). Unrealistic expectations for postpartum sleep are associated both with increased levels of self-reported fatigue (42) and with negative cognitions that have been linked to depression (43). However, parents experiencing postpartum depression and perceiving poor sleep may overestimate the contribution of infant-related sleep disturbance and be encouraged to modify infant sleep behavior in ways that are developmentally inappropriate (44) and may be harmful.

Systematic reviews have indicated that the effectiveness of sleep training to reduce disruption from infant night waking and postpartum fatigue is mixed (12, 45), that sleep training is potentially harmful for infants when implemented prior to 6 months (45), causes parental distress (46), and is associated with concerns regarding infant mental health, development and attachment (47). If, as we have seen here, subjectively reported and objectively measured maternal and infant sleep do not align with parental perceptions of sleep or predict postpartum depression symptomatology, sleep training may be an ineffective use of limited resources (individual or governmental (47)) when implemented for this purpose. Rather than attempting to ameliorate an “infant sleep problem” that may not exist, we might approach negative maternal perceptions of infant sleep with parental support, education, and help to minimize negative thinking (catastrophizing) about sleep and fatigue (48, 49). The management of expectations of new parents about postpartum sleep may help to minimize frustration and sidestep parental crises of self-efficacy (50).

### 4.4. Sleep and postpartum depression by feeding method: Bivariate analyses

With respect to the relationship between infant feeding method and postpartum depression development, our results add to previous findings (51, 52) that run counter to the popular discourse that stress and lack of sleep from breastfeeding (particularly night-time breastfeeding) is likely to cause postpartum depression (30) and that, therefore, the introduction of formula is a form of “self-care” that is beneficial to maternal mental health (31). We found no difference in the level of postpartum depression symptoms or likelihood of postpartum depression risk between exclusively breastfeeding and exclusively formula-feeding women at six, 12 or 18 weeks. Borra and colleagues have argued that breastfeeding does not have a straightforward relationship with the development of depression; they found that breastfeeding as planned decreases the risk of postpartum depression, while being unable to breastfeed as planned increases the risk (53).

An evidence-based understanding of the impact of breastfeeding will be important to consider in further work that aims to prevent postpartum depression. Infant sleep training interventions that aim to limit infant night waking do not take into consideration the evolved physiology of lactation; night-weaning interrupts the dyadic feedback relationship between mother and infant that is necessary to maintain breastmilk supply (54). In other interventions, women are encouraged to use formula so that someone else can feed the infant at night, with the implication that they will be better able to rest, will experience less fatigue, and will therefore avoid postpartum depression (51). Since formula supplementation has been shown to decrease breast milk production and breastfeeding duration (55), interventions that encourage overnight use of formula as a means to prevent postpartum depression may actually *increase* the risks for women who planned to breastfeed but find their milk supply dwindling. The exclusively breastfeeding women in our study were successfully meeting their own breastfeeding goals, which may have been protective against depression symptoms.

### 4.5. Predictors of postpartum depression by feeding method

Our results provide evidence of distinct pathways predicting depression symptomatology for exclusively breastfeeding and exclusively formula-feeding women, despite there being no mean difference in the degree of depression symptomatology between the two groups. At 6 weeks the sole predictor of depression symptomatology for exclusively formula-feeding women was maternal perception of sleepiness. Exclusively breastfeeding women’s depression symptomatology at 6 weeks was not predicted by maternal sleepiness, GSDS, nor by any other objective or subjective sleep parameter variable considered in this study. It is possible that data about women’s history of depression in pregnancy or earlier in life might have altered the predictive relationship for exclusively formula-feeding women, or may have been a predictor of EPDS for exclusively breastfeeding women. Nonetheless, it is noteworthy that sleep measures did not predict postpartum depression symptomatology for women in either feeding group.

At 12 weeks, 6-week EPDS was the sole predictor of depression symptomatology for women in both feeding groups. Sleep measures and reports were not associated with depression symptomatology for either feeding group.

At 18 weeks, however, the predictive pathways for the two groups were again distinct. As discussed in section 4.3, for the full sample 12-week depression symptomatology and 18-week GSDS both emerged in a model that strongly predicted depression symptomatology at 18 weeks. When analyzed by feeding method, however, our results suggest that each predictor was predictive for only part of the sample: the association with prior EPDS was predictive only for exclusively formula-feeding women, as at 12-weeks. For exclusively breastfeeding women, the combination of GSDS and household income strongly predicted depression symptomatology at 18 weeks. This points toward a different mechanism at work than at earlier time points, potentially related to the specific challenges that breastfeeding women face upon return to work. Many exclusively breastfeeding women feel pressure to night wean and/or sleep train as their maternity leave comes to an end, with the aim of being more alert and able to function in a work environment (56). Less affluent exclusively breastfeeding women may feel particularly anxious when approaching the end of subsidized maternity leave without the financial resources to extend it (57).

### 4.6. Study limitations

The study had several limitations. Collecting data through time during the early weeks postpartum and in the home environment presents unavoidable difficulties. Our relatively small sample size (*n* = 61) was further constrained as individual participants were missing data for some measures at some time points. Sleep data were collected for one overnight period at each time point, rather than multiple nights. In a study that occurred very early in the postpartum period, that gathered data from both mothers and infants, and that involved repeated data collection periods, requiring multiple nights of data collection at each time point was felt to be an excessive burden for participants, from the perspective of research ethics. Our sample was homogenous in nature. Almost all participants were White and UK-born, with more than 70% of participants reporting a household income higher than the 2013 UK median income. Recruitment of lower income participants is notoriously difficult (55) and this was likely exacerbated for our study by the intensive data collection and its timing during the first 4 months postpartum. The homogeneity of our sample reflects quite accurately the ethnic and country of origin composition of the region from which we recruited participants. We recruited a slightly higher percentage of non-UK born participants (12% vs. 8%) and a slightly lower percentage of participants of ethnicities other than White (8% vs. 12%) than in the regional population (58). Caution in generalizing these findings to populations that show a greater diversity of economic status, place of birth and ethnicity is warranted. The lack of information about depression during pregnancy and/or previous history of major depression may have limited the predictive power of the regression models constructed at the six-week time point, whereas at 12 and 18 weeks the previous EPDS score was available to include in the model. In future studies of postpartum depression it will be important to ensure that data regarding participants’ mental health history are collected.

## 5. Conclusion

Objective measures and subjective reports of maternal and infant sleep did not correlate with, nor predict, depression symptomology through the first four months postpartum. Women presenting with symptoms of postpartum depression may be more likely to rate their own and their infant’s sleep poorly on retrospective subjective scales, leading to an association between higher depression symptomatology and higher sleep disturbance scores. Approaches that focus on the management of postpartum parental expectations about their own and their infants’ sleep, rather than modification of infant sleep, may be beneficial to reduce parental dissatisfaction and crises of self-efficacy. Women who were exclusively breastfeeding did not experience increased depression symptomatology or show increased likelihood to be at risk of postpartum depression. Breastfeeding does not automatically predispose women to postpartum depression or increase depression symptomatology, and formula feeding is not a feminist “self-care” panacea. We identify distinct pathways shaping postpartum depression experience for women employing different feeding methods. Support for women to meet their breastfeeding goals, as well as structural interventions to extend maternity leave and increase maternity leave pay would more successfully address the predictors of postpartum depression in exclusively breastfeeding women than the introduction of formula.

## Data availability statement

The raw data supporting the conclusions of this article will be made available by the authors, without undue reservation.

## Ethics statement

The studies involving human participants were reviewed and approved by UK National Health Service Research Ethics Committee and Durham University Research Ethics. The participants provided their written informed consent to participate in this study.

## Author contributions

AR and HB contributed to conception and design of the study. AR and LR-S collected and managed the data. AR and FT organized the database and performed the statistical analyses. AR wrote the first draft of the manuscript. FT and HB wrote sections of the manuscript. All authors contributed to manuscript revision, read, and approved the submitted version.

## Funding

This financial support was received from the Durham University International Junior Research Fellowship (COFUND) program, and a Wenner-Gren Foundation for Anthropological Research Post-PhD grant to AR.

## Conflict of interest

The authors declare that the research was conducted in the absence of any commercial or financial relationships that could be construed as a potential conflict of interest.

## Publisher’s note

All claims expressed in this article are solely those of the authors and do not necessarily represent those of their affiliated organizations, or those of the publisher, the editors and the reviewers. Any product that may be evaluated in this article, or claim that may be made by its manufacturer, is not guaranteed or endorsed by the publisher.
